# Evaluation of the Cost-effectiveness of Drug Treatment for Alzheimer Disease in a Simulation Model That Includes Caregiver and Societal Factors

**DOI:** 10.1001/jamanetworkopen.2021.29392

**Published:** 2021-10-22

**Authors:** Kouta Ito, Rick Chapman, Steven D. Pearson, Ali Tafazzoli, Kristine Yaffe, Jerry H. Gurwitz

**Affiliations:** 1 Meyers Primary Care Institute, a joint endeavor of University of Massachusetts Medical School, Reliant Medical Group, and Fallon Health, Worcester; 2 Division of Geriatric Medicine, University of Massachusetts Medical School, Worcester; 3 Institute for Clinical and Economic Review, Boston, Massachusetts; 4 Evidera, Bethesda, Maryland; 5 Department of Psychiatry, Neurology, and Epidemiology and Biostatistics, University of California, San Francisco; 6 San Francisco VA Medical Center, San Francisco, California

## Abstract

**Importance:**

The possibility of widespread use of a novel effective therapy for Alzheimer disease (AD) will present important clinical, policy, and financial challenges.

**Objective:**

To describe how including different patient, caregiver, and societal treatment-related factors affects estimates of the cost-effectiveness of a hypothetical disease-modifying AD treatment.

**Design, Setting, and Participants:**

In this economic evaluation, the Alzheimer Disease Archimedes Condition Event Simulator was used to simulate the prognosis of a hypothetical cohort of patients selected from the Alzheimer Disease Neuroimaging Initiative database who received the diagnosis of mild cognitive impairment (MCI). Scenario analyses that varied costs and quality of life inputs relevant to patients and caregivers were conducted. The analysis was designed and conducted from June 15, 2019, to September 30, 2020.

**Exposures:**

A hypothetical drug that would delay progression to dementia in individuals with MCI compared with usual care.

**Main Outcomes and Measures:**

Incremental cost-effectiveness ratio (ICER), measured by cost per quality-adjusted life-year (QALY) gained.

**Results:**

The model included a simulated cohort of patients who scored between 24 and 30 on the Mini-Mental State Examination and had a global Clinical Dementia Rating scale of 0.5, with a required memory box score of 0.5 or higher, at baseline. Using a health care sector perspective, which included only individual patient health care costs, the ICER for the hypothetical treatment was $183 000 per QALY gained. The result decreased to $173 000 per QALY gained in a traditional societal perspective analysis with the inclusion of patient non–health care costs. The inclusion of estimated caregiver health care costs reduced the ICER somewhat, but the inclusion of QALYs gained by caregivers led to a substantial reduction in the ICER for the hypothetical treatment, to $162 000 per QALY gained in the health sector perspective. In the societal perspective scenario, with the broadest inclusion of patient and caregiver factors, the ICER decreased to $103 000 per added QALY.

**Conclusions and Relevance:**

The findings of this economic evaluation suggest that policy makers should be aware that efforts to estimate and include the effects of AD treatments outside those on patients themselves can affect the results of the cost-effectiveness analyses that often underpin assessments of the value of new treatments. Further research and debate on including these factors in assessments that will inform discussions on fair pricing for new treatments are needed.

## Key Points

**Question:**

How does including caregiver and societal costs affect cost-effectiveness models for the treatment of Alzheimer disease?

**Findings:**

In this economic evaluation, the incremental cost-effectiveness ratios for a novel disease-modifying therapy for Alzheimer disease, using traditional health care sector and societal perspectives, were $183 000 and $173 000 per quality-adjusted life-year gained, respectively. When caregivers’ productivity costs and quality-of-life effects were included, the cost-effectiveness improved substantially.

**Meaning:**

These findings suggest that including estimates of treatment effects on caregivers is a major consideration in determining the cost-effectiveness of treatments for Alzheimer disease.

## Teaser

This economic evaluation describes how including different patient, caregiver, and societal treatment-related factors affects estimates of the cost-effectiveness of a hypothetical disease-modifying AD treatment.

## Introduction

An estimated 6.2 million Americans currently have Alzheimer disease (AD), with that number projected to increase to 7.2 million in just a few years.^1^ Given the great need for effective and disease-modifying treatments for AD and the number of potential patients,^2^ the cost of widely used new treatments could strain health care budgets, requiring either an increase in overall health care spending or a diversion of health care spending from other areas. To ensure that new efficacious treatments for AD are rapidly adopted for use and access is provided to all patients who could benefit, it will be important to deal with potentially discordant perspectives on how to assess the value and frame the national debate on fair pricing for novel effective AD treatments.^3^

While no new pharmacotherapies for AD have been licensed since 2003,^4^ ongoing developments and advances in AD research raise the possibility of novel disease-modifying therapies in the near future. There is a need to assess the value of such treatments, and cost-effectiveness models can serve as a starting point for setting value-based prices and preparing the health care system to pay for new AD drugs.^5,6,7^

We conducted analyses to explore several questions related to determining the cost-effectiveness of treatments for AD. First, how substantial are the differences in cost-effectiveness results for an AD treatment when analyses include estimates of the impact of treatment on patients’ costs and benefits outside the health care system? Second, how substantial is the difference in cost-effectiveness when analyses include caregiver costs and benefits along with those for patients? Finally, what are the policy implications of the differences in cost-effectiveness results for a hypothetical 1-time AD treatment vs a repeated treatment with the same overall effectiveness? To help delineate some of these issues, we evaluated how different scenarios affect estimates of cost-effectiveness for a hypothetical novel and disease-modifying AD treatment.

## Methods

### Model Overview

The intent of this research was not to develop a de novo best practice economic model but to use an existing model well suited to explore the different scenario analyses in a cost-effectiveness analysis of a treatment in a cohort of patients with mild cognitive impairment (MCI). We selected the AD Archimedes Condition Event (ACE) simulator version 3.2 (Evidera), a Microsoft Excel–based patient-level microsimulation model that has been validated against a well-known AD data set (the Uniform Data Set [UDS] from the US National Alzheimer Coordinating Center [NACC-UDS]) and a recent clinical trial of an amyloid-targeted treatment in patients with MCI or mild dementia due to AD (A Study to Evaluate Safety, Tolerability, and Efficacy of Lecanemab in Subjects With Early Alzheimer's Disease [NCT01767311]).^8,9^ This model allows us to estimate the natural history of individuals from MCI to severe dementia due to AD and the potential effects of disease-modifying treatments on disease progression. It describes disease progression through changes in the underlying biomarkers associated with AD, including measures of amyloid and tau levels, and their connections to the clinical presentation of AD, including various patient-level scales of cognition, behavior, function, and dependence. The relationships among changes on these measures over time are quantified using predictive equations derived from long-term observational data from the AD Neuroimaging Initiative (ADNI).^10^ Per the Common Rule, institutional review board approval and informed consent were not required because the study was secondary data analysis of existing data. This study follows the recommendations of the Consolidated Health Economic Evaluation Reporting Standards (CHEERS) reporting guideline.

### Population

For the current analyses, we simulated the prognosis of a hypothetical cohort of patients selected from the ADNI database who received the diagnosis of MCI. Patients in this cohort scored between 24 and 30 on the Mini-Mental State Examination (MMSE) and a clinician assessment of MCI, at baseline.

### Model Inputs

We conducted scenario analyses that varied specific inputs or components of the model (Table 1). In particular, we explored the association of caregiver economic impacts with the study findings by altering the cost breakdown in the model. Model parameters and assumptions are summarized in this section and described in greater detail in the eAppendix in the Supplement.

**Table 1.** Utility and Cost Inputs for Scenarios With and Without Patient Non–Health Care and Caregiver Factors

Cost or utility	Included factors by scenario					
	Health care sector perspective			Societal perspective		
	A	B	C	D	E	F
Health state utilities						
Patient	○	○	○	○	○	○
Caregiver	×	×	○	×	×	○
Health care costs						
Patient	○	○	○	○	○	○
Caregiver	×	○	○	×	○	○
Non–health care costs						
Patient	×	×	×	○	○	○
Productivity costs						
Caregiver	×	×	×	×	○	○

Abbreviations: ○, included; ×, excluded.

#### Disease Progression

The model measured disease progression using interconnected predictive equations for rate of change in biomarkers and clinical scales derived from the ADNI. Published equations from the Assessment of Health Economics in Alzheimer Disease II (AHEAD) model were included in the model to better capture the more advanced stages of dementia.^11,12^ As an individual progressed to more severe stages of dementia, the model triggered a switch from the ADNI equation to the AHEAD equations because the ADNI database did not effectively capture more severe stages of dementia.

#### Disease Severity

AD severity levels are commonly used to predict location of care, mortality, costs of care, and quality of life. Therefore, the model assigned disease severity based on each simulated patient’s characteristics. In the base case, disease severity levels were defined solely based on cognition, specifically the MMSE.^13^

#### Mortality

An equation based on an analysis of data from the Consortium to Establish a Registry for Alzheimer Disease (CERAD) study was used to determine the patient’s risk of death.^12^ It was adjusted by a patient’s age, gender, baseline MMSE score, and the annual rate of MMSE decline.

#### Location of Care

The risk of transition from community care to residential care was determined by the time an individual spent at a particular severity level. This risk was subsequently adjusted by the individual’s current age and gender based on the CERAD study.^14^

#### Hypothetical Treatment Effectiveness

We did not base the inputs for treatment safety or effectiveness on any existing or emerging AD treatment. Instead, we simulated a hypothetical drug that would delay progression to dementia in individuals with MCI, basing the effectiveness of the drug on changes in the CDR–Sum of Boxes (SB) scale, which is currently the most widely used outcome for trials of early AD.^15^ In our base case, changes to CDR-SB were modeled via decreases in the imaging biomarker florbetapir F18 (18F-AV-45) that would correspond to a 25% reduction in the change in CDR-SB scores at 1.5 years after treatment initiation.^16^ This level of change in CDR-SB was based on a previously adopted threshold in a sample size calculation for predementia trials and was subsequently validated by our clinical experts. After consulting with clinical experts, we assumed that the drug would be discontinued once an individual developed moderate dementia, with no residual benefit from treatment beyond discontinuation. Consistent with the adherence observed in clinical trials, a 10% per year discontinuation rate was assumed. The model compared patients allocated to the hypothetical drug therapy vs the same patients without that treatment (ie, usual care/existing therapy).

#### Health State Utilities

The model assigned utility values to reflect patients’ health-related quality of life with different levels of AD (Table 2).^17,18,19,20^ They were estimated from surveys of the general public and caregivers, using the EuroQol–5 Dimension (EQ-5D) questionnaire, a widely used standardized generic instrument.^17,18,21^ Utility values were modified by various factors that were captured in the model, including patients’ cognition, behavior, and institutionalization status, and were used to generate quality-adjusted life-years (QALYs). Health state utility values for patients with dementia and MCI were derived from AD patients’ ED-5D scores, while utility values for caregivers were obtained from EQ-5D scores of informal caregivers of patients with dementia due to AD. We explored the consequences of including caregiver utility by simply adding it to the total patient QALYs gained in the model. After consulting with clinical experts and patient groups, we assumed that caregiver utility returned to the utility of the general population specific to their age when AD patients were institutionalized.

**Table 2.** Health State Utilities and Costs

Utility or cost	Value	Reference
MCI		
Patients	Regression equation^a^	Sullivan et al,^18^ 2006
Caregivers	1.00	Reed et al,^17^ 2017
Dementia		
Patients	Regression equation^a^	Sullivan et al,^18^ 2006
Caregivers		
MMSE 21-26	0.85	Reed et al,^17^ 2017
MMSE 15-20	0.84	Reed et al,^17^ 2017
MMSE 0-14	0.82	Reed et al,^17^ 2017
Costs, US $/mo		
MCI		
Patient health care	1174	Wimo et al,^19^ 2013; Neumann et al,^20^ 2016
Patient non–health care	207	Wimo et al,^19^ 2013; Neumann et al,^20^ 2016
Caregiver health care	705	Wimo et al,^19^ 2013; Neumann et al,^20^ 2016
Caregiver productivity	925	Wimo et al,^19^ 2013; Neumann et al,^20^ 2016
Mild dementia		
Patient health care	1377	Wimo et al,^19^ 2013; Neumann et al,^20^ 2016
Patient non–health care	384	Wimo et al,^19^ 2013; Neumann et al,^20^ 2016
Caregiver health care	731	Wimo et al,^19^ 2013; Neumann et al,^20^ 2016
Caregiver productivity	2044	Wimo et al,^19^ 2013; Neumann et al,^20^ 2016
Moderate dementia		
Patient health care	1833	Wimo et al,^19^ 2013; Neumann et al,^20^ 2016
Patient non–health care	611	Wimo et al,^19^ 2013; Neumann et al,^20^ 2016
Caregiver health care	748	Wimo et al,^19^ 2013; Neumann et al,^20^ 2016
Caregiver productivity	3019	Wimo et al,^19^ 2013; Neumann et al,^20^ 2016
Severe dementia		
Patient health care	2105	Wimo et al,^19^ 2013; Neumann et al,^20^ 2016
Patient non–health care	1025	Wimo et al,^19^ 2013; Neumann et al,^20^ 2016
Caregiver health care	759	Wimo et al,^19^ 2013; Neumann et al,^20^ 2016
Caregiver productivity	5055	Wimo et al,^19^ 2013; Neumann et al,^20^ 2016

Abbreviations: MCI, mild cognitive impairment; MMSE, Mini-Mental State Examination.

^a^ Calculated using a regression equation: utility = 0.408 + 0.010 × (MMSE − 0.04) × (NPI-0.159) × (institutionalized + 0.051) × caregiver. MMSE represents the patient’s current MMSE score. NPI represents the patient’s current Neuropsychiatric Inventory. Institutionalized and caregiver are dummy variables for whether the individual is institutionalized or lives with their caregiver.

#### Costs

We assumed $16 000/year as the cost of the hypothetical drug, based on the median average wholesale price of specialty drugs for chronic medical conditions that were approved in the last 20 years by the US Food and Drug Administration.^22^ Costs for individuals with MCI and dementia were calculated by considering the following cost components (Table 2): patient health care costs (ie, medications, hospitalizations, emergency department visits, outpatient visits, and neuropsychological assessments); patient non–health care costs (ie, dependent living accommodations, community services, consumable goods, and financial support received); caregiver health care costs (ie, medications, hospitalizations, emergency department visits, and outpatient visits); and caregiver productivity costs (ie, caregiver time caring for the patient or work lost by taking care of the patient, whichever value was higher). Costs for those with MCI and mild dementia were taken from a US-based, prospective, longitudinal cohort study of patients with clinician-diagnosed early AD seeking routine care for memory concerns (A Study of Health Care Use and Costs in Participants With Early Stage Alzheimer's Disease [GERAS-US; NCT02951598]).^23^ Costs for those with moderate or severe dementia were extrapolated based on the ratios of costs reported in a prospective, longitudinal cohort study of patients with AD in 3 European countries (GERAS).^19^ In the GERAS studies, hours spent by caregivers were calculated as hours spent caring for the patient or hours of work lost due to caring for the patient, with the higher value applied to the cost calculation. The cost unit for caregiver time was calculated using the opportunity costs approach, ie, by summing lost productive hours and multiplying them by the national average annual gross hourly wage for workers and by lost leisure time for nonworkers (35% of hourly wage for workers). Caregiver non–health care costs were not included due to the lack of relevant data. Patient productivity costs were not included because our target populations were less likely to work for pay.

### Statistical Analysis

To explore the first 2 research questions, we estimated the incremental cost per QALY gained under various scenarios, each of which was designed to include a different range of costs and utilities across patients and caregivers (Table 1). We applied the definitions of perspectives by Kim et al.^24^ The health care sector perspective accounts for any monetary costs of health care, regardless of who bears those costs. The societal perspective accounts for cost components beyond the health care sector perspective, including non–health care costs and productivity loss. To explore research question 3, a separate set of scenarios examined how cost-effectiveness changed when assuming the treatment was administered as a 1-time therapy with varying prices (ie, 1-time price of $100 000 or $250 000) and relative effectiveness (ie, 25% or 50% of the base-case estimate). Cost-effectiveness was estimated using incremental cost-effectiveness ratios (ICERs), comparing the hypothetical drug therapy with usual/existing care. Following standard economic practice, costs and outcomes were discounted at 3% per year over a lifetime horizon.^20^ Data analysis was conducted in the AD ACE simulator version 3.2 (Evidera).

## Results

### Health Care Sector vs Societal Perspectives

Table 3 summarizes the ICERs for the hypothetical drug therapy in various scenarios. The ICER for the hypothetical treatment was $183 000 per QALY gained using the health care sector perspective, which included only patient clinical outcomes and health care costs (scenario A). Patients who received the hypothetical treatment lived an additional 0.193 QALYs (or 0.00 life-years) and incurred total costs of $35 205 by increasing drug costs by $65 735 and decreasing patient health care costs by $30 530. The ICER for the hypothetical treatment decreased to $173 000 per QALY gained in the traditional societal perspective analysis with the inclusion of patient non–health care costs (scenario D).

**Table 3.** ICER for the Hypothetical Drug Therapy From Different Analytic Perspectives

Outcome	$/QALY by scenario^a^					
	Health care sector perspective			Societal perspective		
	A	B	C	D	E	F
ICER	183 000	172 000	162 000	173 000	110 000	103 000

Abbreviations: ICER, incremental cost-effectiveness ratio; QALY, quality-adjusted life-year.

^a^ For a description of which elements are included in each scenario, see Table 1.

### Patient-Only vs Patient and Caregiver Costs

The inclusion of the caregiver’s health care costs also led to a small reduction in the ICER for the hypothetical treatment to $172 000 per QALY gained (scenario B). In contrast, including estimated QALYs gained by caregivers further reduced the ICER for hypothetical treatment to $162 000 per QALY gained (scenario C). When using the broadest societal perspective, with all estimated caregiver productivity and QALY gains included, the ICER decreased to $103 000 per QALY gained (scenario F).

### One-Time vs Repeated Treatment

We assumed a price of $100 000 for the 1-time treatment with the same long-term effectiveness as our repeatedly administered treatment costing $16 000/year (Table 4). The ICER at this price was $361 000/QALY gained, nearly indistinguishable from the base-case finding of $183 000/QALY gained for the repeatedly administered treatment.

**Table 4.** ICER for the Hypothetical Drug Therapy by Different Assumptions About Drug Administration and Price

Cost	ICER, $/QALY
Base case	183 000
One-time treatment, 25% effective, $	
100 000	361 000
250 000	1 140 000
One-time treatment, 50% effective, $	
100 000	265 000
250 000	866 000

Abbreviations: ICER, incremental cost-effectiveness ratio; QALY, quality-adjusted life-year.

## Discussion

There are many controversies regarding the methods of modeling the cost-effectiveness of treatments for AD,^25^ including whether disease measures adequately capture benefits to patients,^26^ how to link short-term measures of cognition with disease progression and the need for institutional care,^27^ how best to capture health-related quality of life,^28^ and whether and how to include the health and cost effects of treatment on caregivers and society.^29^ It is, however, well established that unpaid and informal care for AD patients by family members and other caregivers is widespread, and caregivers may experience adverse emotional and financial consequences leading to decreased quality of life and increased health care costs.^1^ Our model scenarios sought a more comprehensive assessment of how including different elements of health benefits and costs for both patients and caregivers affected cost-effectiveness estimates.^12,25^ The results differed somewhat when the base-case model was expanded to include estimates of the benefits of a hypothetical treatment on caregiver health care costs (scenario B). However, including estimates of the treatment’s impact on caregivers’ quality of life (scenarios C and F) and productivity (scenarios E and F) substantially improved the assessed cost-effectiveness of treatment, reducing the ICER by more than 43%. It is important to note that there is no single best model for evaluating the cost-effectiveness of treatments for AD. We selected the AD ACE model because it is well known in the AD research community and has the ability to evaluate various scenarios among a cohort of people with MCI. But this model, like all others, requires assumptions and has other limitations regarding how its structure translates treatment effect estimates into improvements in quality of life. No matter which model is used, there remain principled arguments about whether including caregiver effects should be the primary perspective taken when evaluating the cost-effectiveness of treatments for AD.^29^

While the inclusion of caregiver costs is relatively straightforward, methods for the inclusion of caregiver quality of life or utility are less well established. In common practice, cost-effectiveness analysis is used to inform decisions by comparing results to cost-effectiveness thresholds related to effects and costs for the patient within the health system. But broadening the unit of analysis to include caregivers presents dilemmas for how to include multiple caregivers, how to combine effects and costs of caregivers and patients, and whether the same opportunity cost threshold should apply when caregivers are included.^30,31,32,33,34,35^ Although the application of different cost-effectiveness thresholds from a health care and a societal perspective is uncommon in published cost-effectiveness analyses, it has been mentioned that using a different threshold according to perspective might be more appropriate.^36^ The widely varying results seen in our scenarios highlight this concern, raising the question of whether a perspective inclusive of caregiver effects should be compared with a different (ie, lower) threshold. There is also an argument about the need for a different cost-effectiveness threshold for severe diseases and conditions, such as AD. Recent literature^37^ has suggested a cost-effectiveness of 5 times annual per capita consumption ($50 000-$80 000), implying that a range of $250 000 to $400 000 could be appropriate.

Although including caregiver effects led to substantial differences in cost-effectiveness results, it is perhaps surprising to find that the same was not true when non–health care costs for patients were included (scenario D). An important question is whether unpaid labor should be valued at the same level as paid labor and whether any value should be attached to time that is not spent at labor, whether paid or unpaid.^20^ This is an especially important issue for diseases such as AD that affect older people who are near or beyond retirement age. It has been argued that value should be accorded to such time,^38,39^ but the appropriate methods and values are not well established. Another question that will arise regarding the comprehensiveness of modeling for AD treatments is whether to include the outcomes related to screening and diagnostic tests. It is a limitation of our study that we did not explore the potential association between cost-effectiveness and varying levels of diagnostic accuracy and cost. Our analyses also found that a 1-time treatment priced at $100 000 had a higher cost-effectiveness ratio than a repeatedly administered treatment priced at $16 000 per year. There may be risks to using cost-effectiveness analysis to value 1-time treatments if those analyses produce unrealistically high prices that must be paid up-front despite significant uncertainty regarding the magnitude of long-term benefits.^40^

Looking forward, the United States continues to support major efforts to develop AD therapeutics that can modify the course of the disease. The day a new treatment is approved for use will be a day of celebration. An effective treatment will be used by millions of US residents; however, this treatment will be very expensive, if not for the individual patient, for the population, potentially putting great strain on health budgets and insurance costs. The use of formal cost-effectiveness analysis will play an influential role informing the public debate on the appropriate price for an AD treatment.^6,41^

### Limitations

There are several limitations to our analyses. First, findings from the ADNI cohort and other model parameters used in the AD ACE may not be generalizable to the current population envisioned for treatment. The ADNI cohort’s participants are predominantly White and generally healthier and more educated than the general population. One study showed that a significant proportion of the estimates of associations among predictors, cognitive performance, and neuroimaging studies differed between the ADNI cohort and a community-based sample.^42^ In addition, the simulated trajectories for CDR-SB in the AD ACE model have not been validated against the community sample, but they do agree well with the mean trajectories from the NACC data set.^9^ Another limitation is that our analyses are based on changes in a single measure of clinical outcome (ie, CDR-SB). Any single measure may not capture the full clinical value of treatment to patients and their families, who care about function, behavioral symptoms, and quality of life; therefore, it is important to emphasize that “there is currently no consensus on what constitutes a clinically meaningful signal of benefit in AD therapeutics.”^43^ Another limitation is that our scenarios focused on the QALY as the only summary measure of health gain. While the QALY has been considered the criterion standard for cost-effectiveness analyses of health care interventions for several decades, some have argued that it should be augmented by the addition of several novel elements of value, such as insurance value or the value of hope.^44^ Some of these novel value elements may be considered especially relevant for conditions like AD. However, at this time, there is no clear consensus on whether it is appropriate to incorporate these elements into cost-effectiveness analyses. An additional limitation of our study is that we did not include analyses of differing adherence with patient and caregiver outcomes.

## Conclusions

In this economic evaluation, the ICER for a hypothetical AD treatment decreased substantially when caregiver costs and utilities were included. What is clear from our analyses is that the inclusion or exclusion of the estimated effects of AD treatments on individuals other than the patients themselves has major consequences for how cost-effective the treatment is judged to be. Whether the estimates of these caregiver effects used in our modeling are accurate or not, the change in cost-effectiveness results implies that modelers should attempt to perform similar scenario analyses for the modeling of any AD treatment. Importantly, federal funding agencies, clinical researchers, life science companies, and regulators should require that data on caregiver effects be gathered as core outcome measures in developmental trials of all AD treatments. Better data are needed with which to determine these effects, and ultimately, policy makers and the public should be made aware of the potential importance—and the potential limitations—of analyses using a broader societal perspective when judging the value and fair price for novel AD treatments.

## Notes

Supplement. **eAppendix.** Supplementary Methods
eReferences.
