# Errors in Simulation Model

**DOI:** 10.1001/jamanetworkopen.2024.33754

**Published:** 2024-08-16

In the Original Investigation titled “Evaluation of the Cost-effectiveness of Drug Treatment for Alzheimer Disease in a Simulation Model That Includes Caregiver and Societal Factors,”^1^ published on October 21, 2021, the authors have reported errors in some of the equations included in the simulation model. For the beta-amyloid load (AV45 level), a negative sign was erroneously reversed to positive. Correction of this error changes some of the estimated costs and incremental cost-effectiveness ratios. There was also an error in the originally reported population, which was described as a “hypothetical cohort of patients selected from the ADNI database who received the diagnosis of MCI. Patients in this cohort scored between 24 and 30 on the Mini-Mental State Examination (MMSE) and had a global Clinical Dementia Rating scale (CDR) of 0.5, with a required memory box score of 0.5 or higher, at baseline… [and] an abnormal memory function score on the Wechsler Memory Scale.” Applying these criteria in the model selects 870 patient records instead of the 873 originally reported in the Supplement of the original article. In addition, a discontinuation rate of 0% was originally reported, but in the corrected the model, this was set to 10% annually. Finally, 2 of the references for health state utility and costs were incorrect. These errors affect the Key Points, Abstract, main text, Tables 2 to 4, and the Supplement. Correcting for these errors does not affect the interpretations or conclusions if the study. This article has been corrected,^1^ and the authors have provided an explanation for the errors and corrections in a Comment posted with the article.^2^
