# Supplementary material for: Evaluation of the Cost-effectiveness of Drug Treatment for Alzheimer Disease in a Simulation Model That Includes Caregiver and Societal Factors
Source: JAMA Netw Open. 2021 Oct 22;4(10):e2129392. doi: 10.1001/jamanetworkopen.2021.29392 (PMC8536950; doi:10.1001/jamanetworkopen.2021.29392)
Supplement: Supplement. — eAppendix. Supplementary Methods eReferences. [file jamanetwopen-e2129392-s001.pdf]

Supplemental Online Content

Ito K, Chapman R, Pearson SD, Tafazzoli A, Yaffe K, Gurwitz JH. Evaluation of the cost-effectiveness of drug treatment for Alzheimer disease in a simulation model that includes caregiver and societal factors. *JAMA Netw Open.* 2021;4(10):e2129392. doi:10.1001/jamanetworkopen.2021.29392

eAppendix. Supplementary Methods

eReferences.

This supplemental material has been provided by the authors to give readers additional information about their work.

© 2021 Ito K et al. *JAMA Network Open.*

Methods

1. Overview and Model Structure

The Alzheimer's Disease Archimedes Condition Event (AD ACE) simulator, a Microsoft Excel-based discretely integrated condition event (DICE) simulation, was used to predict natural history of individuals from preclinical AD to severe dementia due to AD and estimate the effects of disease-modifying treatments (DMTs) on disease progression.[1, 2] The figure below depicts an influence diagram of the key relationships in the model.

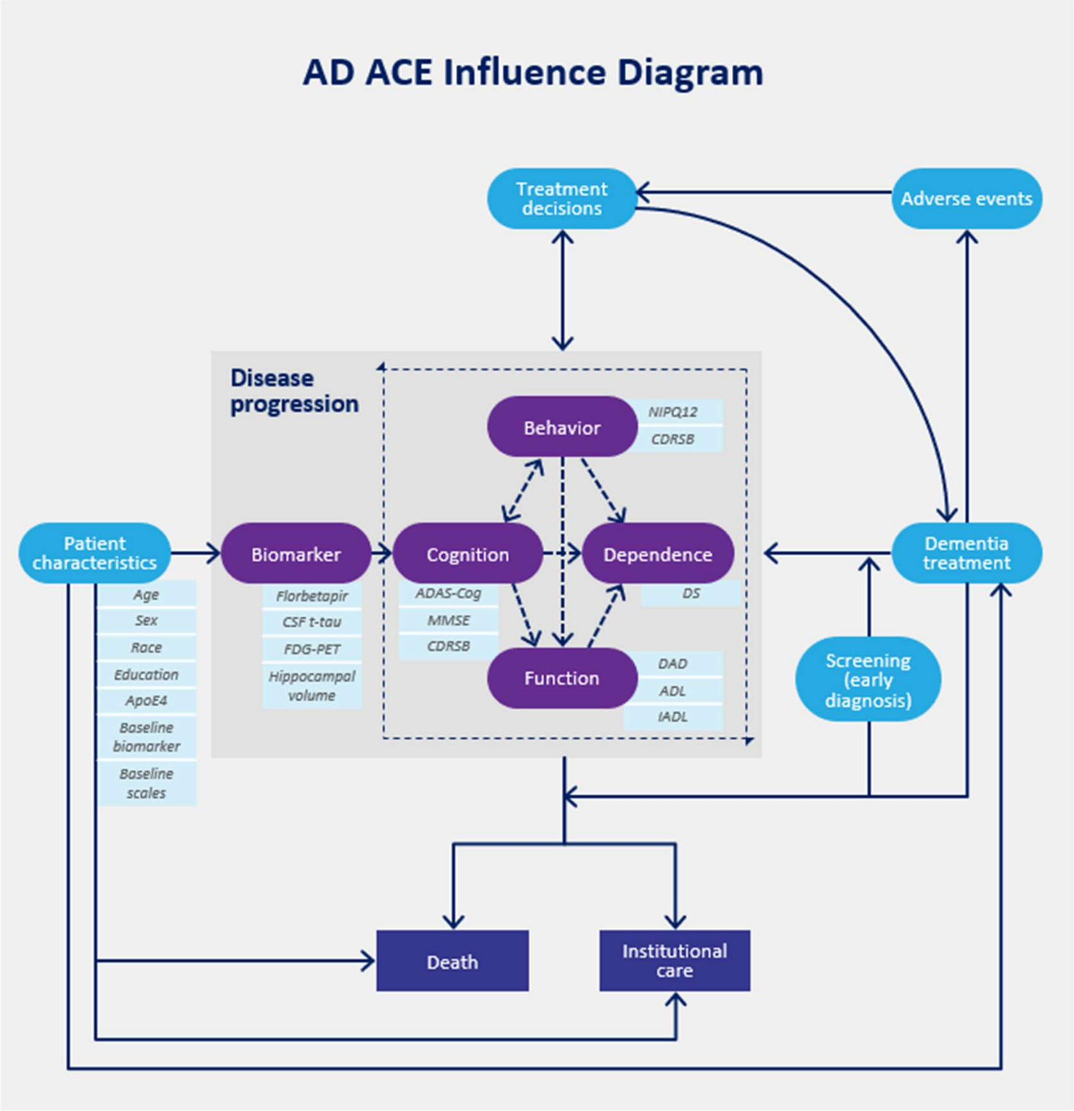

Figure. Influence diagram depicting the key relationships in the AD ACE simulator[1]

ADAS-Cog13, Alzheimer's Disease Assessment Scale-Cognitive Subscale 13; ADL, Activities of Daily Living; CDR-SB, Clinical Dementia Rating Sum of Boxes; CSF t-tau, Cerebrospinal Fluid total-tau; DAD, Disability Assessment scale for Dementia; DS, Dependence Scale; FGD-PET, Fluorodeoxyglucose-positron emission tomography; IADL, Instrumental Activities of Daily Living; MMSE, Mini-Mental State Examination; NPIQ12, Neuropsychiatric Inventory Questionnaire

© 2021 Ito K et al. *JAMA Network Open.*

The AD ACE fully considers interrelated clinical, epidemiologic, and economic outcomes. It incorporates measures of the underlying pathophysiology of AD, including measures of amyloid PET (AV45) and tau (CSF t-tau) levels and their connections to clinical presentation of AD. The relationship between changes on these measures over time is quantified using predictive equations derived from long-term observational data from the Alzheimer's Disease Neuroimaging Initiative (ADNI) to describe disease progression through the evolution of AD biomarkers and various patient-level scales of cognition, behavior, function, and dependence.[3] Prediction of biomarker progression is mainly determined by the patient's characteristics (i.e., age, race, sex, education, and apolipoprotein E4 level) and other biomarkers. Cognitive, behavioral, functional, and dependence scales are, in turn, predicted based on patient characteristics, biomarkers, and other clinical scales. In particular, cognition and behavior influences function and dependence, and function contributes to predicted changes in dependence. The AD ACE represents the course of AD as a combination of evolving conditions and key events using the DICE framework. Different aspects of patient characteristics, patient-level biomarkers and clinical scales, and treatment are defined as conditions that are tracked throughout a patient simulation. At the start of a patient simulation, an initial value is assigned to each condition. These conditions may remain at their initial values or change over time as the simulation continues. Changes in the values of these conditions can affect the occurrence of various events. In the AD ACE, events are defined as instantaneous actions, such as death, institutionalization, and treatment start/switch. Multiple events can occur simultaneously. Disease progression determines a patient's quality of life, risk of institutionalization, societal costs of care, and mortality. The primary outputs of the AD ACE model are quality-adjusted life years (QALYs), total life years, disease management costs, and incremental cost-effectiveness ratios. The design of the AD ACE was based on a systematic literature review of AD economic modelling, the Modeling Good Research Practices disseminated by the International Society for Pharmacoeconomics and Outcomes Research, and a review of ongoing clinical trials for both symptomatic treatments and DMTs of AD.[4, 5]

2. Key Model Choices and Assumptions
Below is a list of key model choices (Table 1).

**Table 1. Key model assumptions**	
**Assumption**	**Rationale**
Hypothetical drug therapy will be discontinued once an individual developed moderate dementia, with no residual benefit from treatment beyond discontinuation	Clinical experts considered this to be a reasonable assumption.
Hypothetical drug therapy will reduce the decline in CDR-SB by 25% during the course of therapy.	Clinical experts considered this to be a reasonable assumption.
Hypothetical drug therapy does not directly affect mortality or institutionalization beyond its impact on clinical scale measures.	Clinical experts considered this to be a reasonable assumption.
10% per annum discontinue hypothetical drug therapy.	Assumptions is consistent with discontinuation rates observed in clinical trials.
Adverse event-related costs and disutility associated with hypothetical drug therapy are not included in the model.	Assumptions would be arbitrary due to the hypothetical nature of the therapeutic intervention.
Individuals with MCI will not enter a nursing	They are functionally independent by our

© 2021 Ito K et al. *JAMA Network Open.*

home.	definitions.
Individuals who enter a nursing home do not return to community living.	Clinical experts considered this to be a reasonable assumption.
Caregivers of individuals with MCI will experience no utility loss.	Clinical experts considered this to be a reasonable assumption.
Patient indirect cost (i.e., value of lost production time) will not be considered.	Clinical experts considered this to be a reasonable assumption.
Suboptimal management of comorbid conditions is captured by a patient's increased risk of institutionalization, mortality, and societal costs of care and decreased quality of life.	Clinical experts considered this to be a reasonable assumption.

CDR-SB, Clinical Dementia Rating Sum of Boxes; MCI, mild cognitive impairment

3. Population

We targeted individuals with diagnosis of MCI, using inclusion criteria for MCI based on the ADNI. We extracted participants with early and late MCI from the ADNI cohort to define the MCI group. The inclusion criteria for MCI in ADNI were:

- Subjective memory concern as reported by participant, study partner, or clinician
- Abnormal memory function score on Wechsler Memory Scale; MMSE score of 24-30; CDR = 0.5; Memory Box score = 0.5 or higher
- General cognition and functional performance such that a diagnosis of AD cannot be made by the site physician
- Cholinesterase inhibitors and memantine are allowable if stable for 12 weeks prior to screening

The AD ACE produced the baseline characteristics of individuals with MCI (Table 2).

Table 2. Baseline characteristics of individuals with MCI (N=873)

**Parameter**	**Mean**	**SD**
Age	72.97	7.66
MMSE	27.59	1.81
ADAS-Cog13	16.52	6.79
CDR-SB	1.52	0.89
NPI-Q12	2.01	2.88
DAD	84.75	8.00
ADL	64.90	0.00
IADL	14.70	0.00
DS	3.83	0.81
Proportion male	59.0%	Not available
Proportion ApoE4 positive	49.9%	Not available
CDR-SB progression rate in 1.5 years	0.46	0.45

MMSE, Mini-Mental State Examination; ADAS-Cog13, Alzheimer's Disease Assessment Scale-Cognitive Subscale 13; CDR-SB, Clinical Dementia Rating Sum of Boxes; NPI-Q12, Neuropsychiatric Inventory Questionnaire 12; DAD, Disability Assessment scale for Dementia; ADL, Activities of Daily Living, IADL, Instrumental Activities of Daily Living; DS, Dependence Scale; SD, standard deviation

4. Interventions

We hypothesized a drug that will alter the course of AD in individuals with MCI. In our base

© 2021 Ito K et al. *JAMA Network Open.*

case, this was modeled via a reduction in CDR-SB decline of 25%. We assumed that the drug was discontinued once an individual developed moderate dementia, with no residual benefit from treatment beyond discontinuation. The models compared those who are allocated to hypothetical drug therapy with those who are not (i.e., usual care/existing therapy), as well as with different treatment scenarios using alternative assumptions, as described below.

5. Input Parameters

Clinical Inputs

Disease progression

The AD ACE measures disease progression using interconnected predictive equations for rate of change in biomarkers and clinical scales derived from long-term observational data from the ADNI.[3] The ADNI was launched in 2003 with the primary goal of testing whether biomarkers and clinical scales can be combined to measure the progression of MCI and mild dementia due to AD. Longitudinal assessments for the following measures were extracted from a total of 1,735 individuals from the ADNI dataset to derive disease equations using a linear mixed-modeling framework: cerebrospinal fluid proteins (beta amyloid1-42; total-tau); fluorodeoxyglucose-PET (a functional imaging biomarker), and one magnetic resonance imaging measurement of hippocampal volume; as well as three cognition scales (MMSE, CDR-SB, and ADAS-Cog 13) and one behavioral scale (NPIQ12). Published equations from the Assessment of Health Economics in Alzheimer's Disease II (AHEAD) model are further included in the AD ACE to compute individual's functional and dependence scales and to better capture the more advanced stages of dementia than the ADNI database does.[6, 7] As an individual progresses to more severe stages of dementia, the AD ACE triggers a switch from the ADNI equation to the AHEAD equations.

Disease severity

The AD ACE predicts disease progression without relying on disease severity levels directly. However, AD severity levels are commonly used as predictors of location of care, mortality, costs of care, and quality of life. Therefore, AD ACE assigns disease severity based on each simulated patient's characteristics. In our analysis, disease severity levels were solely on cognition (MMSE, CDR-SB, and ADAS-Cog).

Mortality

The presence and severity of MCI and dementia due to AD are associated with reduced survival. A Weibull parametric equation derived from the analysis of data from the Consortium to Establish a Registry for Alzheimer's Disease (CERAD) study was used to determine the patient's risk of death.[7] This equation predicts survival using the individual's age, sex, and baseline MMSE score to set the scale parameter of the Weibull distribution.

$$\text{Weibull shape} = 1.85$$
$$\text{Weibull scale} = 4.60 + 0.11 * \text{Age} - 0.0009 * \text{Age}^2 + 0.33 * \text{Female} + 0.023 * \text{MMSE}$$

Age represents the individual's current age, MMSE represents the individual's baseline MMSE, and Female is a dummy variable for whether the patient is female.

Location of care

In general, the risk of institutionalization increases as an individual progresses to the more severe stages of dementia due to AD. The risk of transition from community care to residential care was linked to the time an individual spent at a particular disease severity level and subsequently adjusted for each individual by applying a hazard ratio modified by the individual's current age and sex based on the CERAD study (Tables 3 and 4).[8]

© 2021 Ito K et al. *JAMA Network Open.*

Table 3. Transition probabilities of institutionalization based on time spent at different disease severity stages				
**Severity of dementia***	**1 years**	**2 years**	**3 years**	**4 years**
Mild	0.032	0.037	0.065	0.063
Moderate	0.118	0.098	0.097	0.118
Severe	0.378	0.200	0.077	0.075

Table 4. Hazard ratios by patient characteristics			
**Severity of dementia***	**Female**	**Age 65-74**	**Age 75+**
Mild	0.65	0.84	1.80
Moderate	0.97	1.09	0.98
Severe	1.21	1.02	0.92

* We will define severity of dementia using CDR-SB, where Mild = 0.5-9.5, Moderate = 9.5-16, and Severe = 16-18.

Effectiveness
The Food and Drug Administration and European Medicine Agency guidelines encourage the use of a single, unitary and valid measure of efficacy including elements of both cognitive and functional performance.[9, 10] To date, however, there is no gold standard for scales that are capable of detecting clinically meaningful change in early AD. Therefore, we used the CDR-SB scale that is currently the most widely used outcome for trials of early AD.[11] We assumed a minimally clinically meaningful treatment effect corresponding to 25% reductions in the annual rates of change in CDR-SB by hypothetical drug therapy.[12]

Health State Utilities
Patient utility
Utilities for individuals with MCI and dementia due to AD was calculated using a published regression equation based on Euro-QOL 5-dimension questionnaire (EQ-5D) scores of patients with AD in Sweden, Denmark, Finland, and Norway:[13]

$$\text{Utility} = 0.408 + 0.010 * \text{MMSE} - 0.04 * \text{NPI} - 0.159 * \text{Institutionalized} + 0.051 * \text{Caregiver},$$

where MMSE represents the patient's current MMSE score, NPI represents the patient's current NPI, And Institutionalized and Caregiver are dummy variables for whether the individual is institutionalized or lives with their caregiver.

Caregiver utility
Caregiver utilities was based on the EQ-5D scores of informal caregivers of individuals with dementia due to AD in France, Germany, and the UK and stratified by the patient's MMSE (Table 5).[14] There are still methodological challenges in incorporating caregiver utility in cost-effectiveness analyses.[15] Currently available caregiver utility does not reflect the dynamic of spillover effects in relation to institutionalization, end-of-life care, and death of patients.[16] We explored the impact of caregiver utility on the study findings by including or excluding it from the AD ACE model.

Table 5. Caregiver utility		
**AD severity by MMSE range**	**Base-case**	**SD**
21-26	0.86	0.18
15-20	0.85	0.19

0-14	0.82	0.23

SD, standard deviation

Cost Inputs
The costs used in the model included the drug cost and the health care and societal costs of care. All costs were inflated to the current year using the Consumer Price Index for Medical Care for All Urban Consumers.[17]

Drug costs
We assumed $16,000 per year as the base-case price of the hypothetical drug, based on the median average wholesale price of specialty drugs for chronic medical conditions that were approved in the last 20 years by the US Food and Drug Administration.[18] This cost was varied in scenario and sensitivity analyses to test a range of prices.

Costs of AD
Community care costs for individuals with MCI and mild dementia were taken from a US-based, prospective, longitudinal cohort study of patients with clinician-diagnosed early AD seeking routine care for memory concerns (GERAS-US).[19] Total health care costs included patients' health care costs. Total societal costs were calculated by adding the following cost components: patient health cate costs (i.e., medications, hospitalizations, emergency department visits, outpatient visits, and neuropsychological assessments); patient non-health care costs (i.e., dependent living accommodations, community services, consumable goods, and financial support received); caregiver health care costs (i.e., medications, hospitalizations, emergency department visits, and outpatient visits); and caregiver productivity costs (i.e., value of lost production time based on the national average wage per US population) (Table 6). Community care costs for individuals with moderate or severe dementia were extrapolated based on the ratios of costs reported in a prospective, longitudinal cohort study of patients with AD in three European countries (GERAS).[20] Due to the older age of our target populations, patient indirect costs (i.e., lost production) were not considered.

Table 6. Community care costs per month

Cost category	MCI	Mild dementia	Moderate dementia	Severe dementia
Patient health care	1,174	1,377	1,833	2,105
Patient non-health care	207	384	611	1,025
Caregiver health care	705	731	748	759
Caregiver productivity	925	2,044	3,019	5,055
Total	3,011	4,535	6,210	8,943

MCI, mild cognitive impairment

Residential care costs were extracted from GERAS and the 2019 Genworth: Cost of Care Survey National Median Costs (Table 7).[21, 22]

Table 7. Residential care costs per month

Residential care costs	8,175

6. Model Outcomes
Model outcomes will include life years gained, QALYs gained, and total costs for each intervention over a lifetime horizon. All costs and QALYs will be reported as discounted values, using a discount rate of 3% per annum.

© 2021 Ito K et al. *JAMA Network Open.*

7. Model Analyses
Cost-effectiveness was estimated using incremental cost-effectiveness ratios, with incremental analyses comparing hypothetical drug therapy to usual/existing therapy, from the societal and healthcare sector perspectives. Cost inputs considered in the analyses will be defined by the selected perspective. Cost inputs considered in the analyses were defined by the selected perspective, as detailed in Table 11. In particular, we will explore the caregiver economic impact on the study findings by altering the cost breakdown in the AD ACE model.

Scenario Analyses
To explore the research questions 1 and 2, we conducted several scenario analyses that vary specific inputs or components of the model (Table 8).

Table 8. Utility and Cost Inputs for Question 1 and 2 Scenarios

Perspective	Health care sector			Societal		
Scenario	A	B	C	D	E	F
**Health state utilities**						
Patient	○	○	○	○	○	○
Caregiver	×	×	○	×	×	○
**Health care costs**						
Patient	○	○	○	○	○	○
Caregiver	×	○	○	×	○	○
**Non-health care costs**						
Patient	×	×	×	○	○	○
**Indirect costs**						
Caregiver	×	×	×	×	○	○

To explore the research question 3, a separate set of scenarios examined the impact of assuming the treatment was administered as a one-time therapy with varying prices (i.e,. one-time price of $100,000 or $200, 000) and relative effectiveness (i.e., 25% like in the base-case estimate and 50%).

8. Model Validation
The results of external validation indicate that the AD ACE could closely match cognitive decline observed in both a well-known AD dataset (i.e., the Uniform Data Set [UDS] from the US National Alzheimer's Coordinating Center [NACC-UDS]) and a recent clinical trial of an amyloid-targeted treatment in subjects with MCI or mild dementia due to AD (i.e., BAN2401-G000-201 trial [study 201]).[23]

© 2021 Ito K et al. *JAMA Network Open.*

eReferences

1. Kansal AR, Tafazzoli A, Ishak KJ, Krotneva, S. Alzheimer's disease Archimedes condition-event simulator: development and validation. Alzheimers Dement (NY). 2018;4:76-88.
2. Caro JJ. Discretely integrated condition event (DICE) simulation for pharmacoeconomics. Pharmacoeconomics. 2016;36(7):665-672.
3. Alzheimer's Disease Neuroimaging Initiative (ADNI). Available at http://adni.loni.usc.edu/. Accessed March 26, 2021.
4. Hernandez L, Oden A, DosSantos R, Getsios D. Systematic review of model-based economic evaluations of treatments for Alzheimer's disease. Pharmacoeconomics. 2016;34(7):681-707.
5. Caro JJ, Briggs AH, Siebert U, et al. Modeling good research practices - overview: a report of the ISPOR-SMDM Modeling Good Research Practices Task Force-1. Value Health. 2002;15(6):796-803.
6. Getsios D, Blume S, Ishak KJ, Maclaine GD. Cost effectiveness of donepezil in the treatment of mild to moderate Alzheimer's disease: a UK evaluation using discrete-event simulation. Pharmacoeconomics. 2010;28(5):411-427.
7. Guo S, Getsios D, Revankar N, et al. Evaluating disease modifying agents: a simulation framework for Alzheimer's disease. Pharmacoeconomics. 2014;32(11):1129-1139.
8. Neumann PJ, Araki SS, Longo AA, et al. Measuring Alzheimer's disease progression with transition probabilities: estimates from CERAD. Neurology. 2001;57(6):957-964.
9. U.S. Department of Health and Human Services, Food and Drug Administration. Center for Drug Evaluation and Research (CDER), Center for Biologics Evaluation. Early Alzheimer's Disease: Developing Drugs For Treatment, Guidance for Industry. 2018. https://www.fda.gov/regulatory-information/search-fda-guidance-documents/alzheimers-disease-developing-drugs-treatment-guidance-industy. Accessed March 26, 2021.
10. Committee for Medicinal Products for Human Use (CHMP). Guideline on the clinical investigation of medicines for the treatment of Alzheimer's disease. 2018. https://www.ema.europa.eu/en/documents/scientific-guideline/guideline-clinical-investigation-medicines-treatment-alzheimers-disease-revision-2_en.pdf. Accessed March 26, 2021.
11. Cummings J, Lee G, Ritter A, Sabbagh M, Zhong K. Alzheimer's disease drug development pipeline: 2020. Alzheimers Dement (NY). 2020;6(1):e12050.
12. Huang Z, Muniz-Terrera G, Tom BDM. Power analysis to detect treatment effects in longitudinal clinical trials for Alzheimer's disease. Alzheimers Dement (NY). 2017;3(3):360-366.
13. Jönsson L, Andreasen N, Kilander L, et al. Patient- and proxy-reported utility in Alzheimer's disease using the EuroQoL. Alzheimer Dis Assoc Disord. 2006;20(1):49-55.
14. Reed C, Barrett A, Lebrec J, et al. How useful is the EQ-5D in assessing the impact of caring for people with Alzheimer's disease? Health Qual Life Outcomes. 2017;15(1):16.
15. Wittenberg E, James JP, Prosser LA. Spillover effects on caregivers' and family members' utility: a systematic review of the literature. Pharmacoeconomics. 2019;37(4):475-499.
16. Lin PJ, D'Cruz B, Leech AA, et al. Family and caregiver spillover effects in cost-utility analyses of Alzheimer's disease interventions. Pharmacoeconomics. 2019;37:597-608.
17. United States Department of Labors. Bureau of Labor Statistics. Consumer Price Index. Available at http://www.bls.gov/cpi/. Accessed March 26, 2021.
18. Hernandez I, Bott SW, Patel AS, et al. Pricing of monoclonal antibody therapies: higher if used for cancer. Am J Manag Care. 2018;24(2):109-112.
19. Robinson RL, Rentz DM, Andrews JS, et al. Cost of early stage Alzheimer's disease in the United States: cross-sectional analysis of a prospective cohort study (GERAS-US). J Alzheimers Dis. 2020;75(2):437-450.

© 2021 Ito K et al. *JAMA Network Open.*

20. Wimo A, Reed CC, Dodel R, et al. The GERAS study: a prospective observational study of costs and resource use in Community dwellers with Alzheimer's disease in three European countries – study design and baseline findings. J Alzheimers Dis. 2013;36(2):385-399.
21. Genworth: Cost of Care Survey 2019 National Median Costs. https://www.genworth.com/aging-and-you/finances/cost-of-care.html. Accessed March 26, 2021.
22. Reed C, Happich M, Argimon JM, et al. What drives country differences in cohort of Alzheimer's disease? An explanation from resource use in the GERAS study. J Alzheimers Dis. 2017;57(3):797-812.
23. Tafazzoli A, Kansal A. Disease simulation in drug development. External validation confirms benefit in decision making. Available at https://www.evidera.com/wp-content/uploads/2018/10/07-Disease-Simulation-in-Drug-Development_Fall2018.pdf. Accessed March 26, 2021.

© 2021 Ito K et al. *JAMA Network Open.*
